# Effects of repetitive mild traumatic brain injury on weight gain and chronic behavioral outcomes in male rats

**DOI:** 10.1371/journal.pone.0287506

**Published:** 2023-07-20

**Authors:** Martha A. Graham, Patria T. Juzang, Todd E. White

**Affiliations:** 1 Center for Visual and Neurocognitive Rehabilitation, Atlanta VA Health Care System, Decatur, Georgia, United States of America; 2 Department of Neurobiology, Morehouse School of Medicine, Atlanta, Georgia, United States of America; University of Florida, UNITED STATES

## Abstract

To assess the long-term behavioral effects of repetitive mild traumatic brain injury (rmTBI), we employed a preclinical model of rmTBI and performed a battery of behavioral tests starting 14 weeks post-injury. Male Sprague-Dawley rats received four unilateral mild (6 m/s; 0.5 mm depth) controlled cortical impacts (CCI), centered 4 mm posterior and 3–4 mm lateral to the bregma, administered at five-day intervals. The animals’ weights were monitored throughout the study. We tested the rats for anxiety-like (elevated plus maze, open field test), depression-like (forced swim test), locomotor (rotarod, open field test), and spatial learning and memory (Morris water maze (MWM)) behavioral deficits. Overall, a mild behavioral phenotype was observed. Significant deficits were observed with the MWM, indicating that our injury model disrupts spatial learning and memory. An interesting aspect of these data is a directional/visual component to the spatial learning and memory deficits dependent on the zone in which the trial began. With the injury being unilateral, there may be an imbalance in visual acuity that contributes to the observed deficits. Analysis of weight gain data demonstrated that rmTBI reduces weight during the period while injuries are occurring. This may represent another measure that can be tracked to determine injury severity and recovery. RNA-seq analysis demonstrated that gene expression at the chronic endpoint could distinguish between the experimental groups even with a mild behavioral phenotype. Future studies would include a more severe injury paradigm to promote longer-lasting behavior changes.

## Introduction

Traumatic brain injury (TBI) occurs when an external force causes damage to the brain and is a significant health concern for both civilian and military populations. In 2014, there were nearly 3 million TBI-related emergency room visits, hospitalizations, and deaths in the United States [1]. Annual TBI-related deaths alone were over 60,000 from 2016–2019 [2, 3]. TBI can range in severity from mild to severe and is often characterized by several physical and cognitive effects, such as unconsciousness and memory loss [4]. TBI is a common consequence of slip and fall and automobile accidents, contact sports (e.g., American football), and injuries related to military service in combat zones (e.g., blast exposure). There were 16,476 reported cases of TBI in 2020 and 439,609 cases since 2000 among active-duty military service members [5]. Mild TBI (mTBI) is the most prevalent form of TBI for civilian and military populations, comprising more than 82% of all cases. The increased diagnosis of chronic traumatic encephalopathy (CTE) in former football players and the increased incidence of TBI among Veterans of the wars in Iraq and Afghanistan has resulted in increased emphasis on the issue in both preclinical and clinical research.

Studies have shown that single mTBI models usually will not produce consistent lasting cognitive and behavioral deficits that are measurable at chronic time points [6, 7]. However, there is evidence that repetitive mTBI (rmTBI) models can accomplish this goal. Several animal models of rmTBI have been shown to elicit cognitive deficits similar to a clinical head injury [6–9]. These models have demonstrated that lateral fluid percussion (LFP) and controlled cortical impact (CCI) injury models can produce cognitive and sensorimotor deficits in rodents up to 18 months post-injury [6–9]. The primary goal of this study was to determine if a model of chronic repetitive mild TBI affects behavioral outcomes in adult male rats in a CCI injury model.

## Materials and methods

### Animals

Twenty-four adult male CD^®^ IGS:001 Sprague-Dawley rats (226–250 g) were purchased (Charles River, Wilmington, MA) and were allowed to acclimate to the Veterinary Medical Unit (VMU) facilities for nine days prior to any experimental procedures. Animals were dual-housed in a temperature-controlled colony room in the VMU and maintained on a 12:12-h light/dark cycle (light on at 6:00 am) with *ad libitum* access to water and standard rat chow. The animals were randomly assigned to one of two groups: SHAM (n = 12) and TBI (n = 12). All experiments were approved by the Atlanta VA Health Care System Institutional Animal Care and Use Committee and were conducted in compliance with the National Institutes of Health Guide for the Care and Use of Laboratory Animals.

### Surgical procedures and injury model

Anesthesia was induced with 5% isoflurane. Sterile-tip surgery was performed on all animals maintained under 3% isoflurane anesthesia. The scalp was shaved, an incision was made, and the skin was retracted. A craniectomy was done with a 6 mm diameter trephan drill bit centered 4 mm posterior and 3–4 mm lateral to bregma (approximately midway between lambda and bregma). All animals received a craniectomy, while only TBI animals received injuries.

Repetitive mild traumatic brain injury (rmTBI) was modeled by performing four unilateral mild CCI injuries administered at five-day intervals. A literature review of similar animal models indicated that this could provide the desired balance between chronic behavioral deficits and minimal overt damage to the brain [6–9]. CCI was rendered via a pneumatic CCI device (Pittsburgh Precision Instruments, Inc., Pittsburgh, PA). Each injury was conducted with a 5 mm diameter impact tip under the following parameters: impact angle of 15° from vertical, 6 m/s velocity, 0.5 mm depth. Successive injuries, on days 5, 10, and 15, were performed by re-exposing the same craniectomy via re-opening the scalp incision and removing accumulated scar tissue and fascia, followed by a CCI with the previously mentioned parameters. Sham animals also had the craniectomy re-exposed in the same manner. The wound cavity was thoroughly cleaned, and bleeding was stopped prior to skin closure with a monofilament suture after each injury. Debridement of the incision was also performed after each injury prior to closure to promote proper wound healing.

### Behavioral testing

Five testing paradigms were chosen to measure anxiety-like and depressive-like behaviors, as well as motor activity and coordination. Testing began 14 weeks post injury and took place over a two-week period. The order of tests was chosen to minimize stress on the animals and to reduce the confounding impact that the more stressful tests may have upon the results from other less stressful paradigms. Elevated plus maze (EPM), rotarod (RR), open field test (OFT), and forced swim test (FST) were performed during the first week of testing. The Morris water maze (MWM) was performed during the second week of testing.

#### Elevated plus maze

The elevated plus maze (EPM) assesses anxiety-like and risk-taking behaviors (Walf and Frye 2007). The apparatus was comprised of four equally sized arms, two open runways and two closed runways (50 cm x 10 cm), constructed in the shape of a plus sign and shrouded with curtains on all sides to prevent external visual confounders (Stoelting, Wood Dale, IL). Animals were placed in the center of the maze and allowed to explore the apparatus for five minutes. The apparatus was cleaned between animals with a diluted 7% isopropyl alcohol solution to reduce potential olfactory trails. EPM tests were tracked with ANY-maze software (Stoelting, Wood Dale, IL) and time spent in both the open and closed arms was measured.

#### Rotarod

The rotarod (RR) test is a commonly used behavioral test for assessing motor acuity (Jones and Roberts 1968). The RR paradigm used for this study was a single-lane independent accelerating apparatus (Omnitech Electronics, Columbus, OH) consisting of a 70 mm diameter rod capable of gradual acceleration. Falls off the rod were detected by infrared photocells. RR tests were performed with an accelerating rod profile, one to 30 maximum revolutions per minute, for a maximum of five minutes. There were four total trials performed, with a 25-minute inter-trial interval between each test. Trials were stopped when the animal fell off the rod but were repeated if the animals fell off in less than 15 seconds. The three trials with the longest latency to fall were considered for analysis.

#### Open field test

The open field test (OFT) is a center-avoidance paradigm that measures anxiety-like behavior (Walsh and Cummins 1976). Animals were placed in a corner of a 43.38 x 43.38 x 30.28 cm chamber (Med Associates, Fairfax, VT) and allowed to explore for five minutes. The apparatus was cleaned between animals with a diluted 7% isopropyl alcohol solution to reduce potential olfactory trails. The center zone of the chamber was defined, and the animals’ movement tracked with ANY-maze software. NOTE: The interior dimensions of the apparatus chamber were likely inadequate to accommodate the considerable size of the animals 14 weeks post-injury. There was not much space for the animals to move around outside of the center zone, and ANY-maze may have mistakenly documented center zone entries and time where an animal may have been proximal to the center zone or straddling the defined border of the zone rather than inside of it. A custom-built apparatus that takes the larger size of the animals into account will be required in future studies to avoid this issue.

#### Morris water maze

The Morris water maze (MWM) is a test for impairments in spatial learning and memory (Morris 1984). The MWM apparatus was a 6 ft diameter galvanized steel tank (Stoelting, Wood Dale, IL) filled with water (28–30°C) dyed with non-toxic black food coloring. The tank was divided into four equal zones–northwest (NW), northeast (NE), southwest (SW), and southeast (SE)–with ANY-maze software with a distal visual cue placed on the center wall of each zone. The platform was placed in the NW zone. MWM testing took place over a 4-day period with each trial beginning at a different start position. The first day consisted of four platform training trials, with the top of the platform 1 inch above the water line. The remaining platform trials were performed with the platform 1 inch below the water line. A total of 20 platform trials were conducted on days two (8 trials), three (8 trials), and four (4 trials). A probe trial was also conducted on day four, where the platform was removed from the tank to assess reference memory. All trials were allowed to take up to 60 s, with the trial ending 3 seconds after the animal successfully located the platform in the platform trials and after 60 seconds in the probe trial. If the animal failed to locate the platform within 60 seconds, the latency to the platform was recorded as 60 seconds. Distance traveled, speed, and time spent in each zone was also measured. Time spent moving towards and away from the platform zone during the probe trial was assessed as well.

#### Forced swim test

The rats were also assessed using the forced swim test (FST). FST data is not included in this paper due because the FST cylinders used for this study were too short to be filled with enough water to prevent larger animals from touching the bottom of the cylinder with their tails. SHAM animals tended to “rest” the end of their tails on the bottom of the cylinder and remain relatively inactive throughout the test. Taller cylinders will be required for future studies to prevent this problem. This information is included so readers are aware of all procedures the animals experienced. Briefly, to understand the exposure of the animals, FST is a behavioral despair paradigm that measures depressive-like behavior in rodents [10]. Animals were placed in a 29.21 x 44.45 cm cylindrical tank (Med Associates, Fairfax, VT) containing water (28–30°C). Each FST consisted of two swims per animal: a 15-minute pre-exposure and a 5-minute final test 24 hours after the pre-exposure.

#### Weight measurements

Animals were weighed immediately prior to each surgical procedure during the intra-op period (four measurements total) and once weekly for seventeen weeks during the post-op period. A final weight measurement was also taken on the day of sacrifice. Three data points were recorded for each weight measurement and consisted of; overall weight, weight gained per time point, and total weight gained.

#### RNA-seq analysis

Total RNA was extracted from whole blood drawn at the time of sacrifice (~16 weeks post-op). RNA libraries were created using the Total RNA workflow (Life Technologies). Libraries were assessed using a Bioanalyzer DNA High Sensitivity chip (Agilent). The libraries were cloned onto sequencing beads and deposited on a 540 sequencing chip. The libraries were then sequenced on the Ion S5™ System. Sequencing read data were aligned to the rat reference genome using STAR and Bowtie2. Transcripts were annotated using the latest RefSeq rn annotation, and novel transcripts were quantified using denovo guides generated by cufflinks. Gene expression and exon expression data were analyzed using partek Genomics Suite (v7.7) and LIMMA (using R). Count data were filtered to remove low expression genes (<10 reads/gene) and low occurrence (present in <50% of samples). If Batch effects were observed, data were prenormalized using CombatSeq. Differential gene and exon expression were determined using linear models, correcting for batching, and using a corrected p <0.05 (FDR). A 1.5-fold change cutoff was used. Genes and exons identified as significantly different with ANOVA were used as classifiers for prediction modeling to identify a panel of genes that best fits/discriminates between experimental groups.

#### Statistical power and sample size

Sample size was determined *a priori* with ClinCalc’s sample size calculator with the following criteria: 1) two independent study groups, 2) primary endpoints are averages, 3) control group mean of 100 with standard deviation of 15, 4) 15% change in experimental group, 5) enrollment ratio of 1, 6) significance level of 0.05, and 7) statistical power of 90%. This calculation was substantiated *post hoc* per Mead’s resource equation for blocked designs, defined as E = N−b−T, where E = degrees of freedom for error, N = degrees of freedom for total sample size, b = degrees of freedom for blocks, and T = degrees of freedom for treatments [11]. An E value between 10 and 20 indicates ideal use of resources, E<10 indicates inadequate resources, and E>20 indicates ample resources [11]. The resource equation for this study was E = 23−1−1 = 21; thus, we confirmed that an appropriate *n*–value (12) was selected for this study.

#### Statistical analysis

Behavioral figures and tabular data were generated with Microsoft Excel (Microsoft Corporation, Redmond, WA). Data are expressed as the mean (M) ± standard error of the mean (SEM). Groups were compared via Student’s t-test for EPM, OFT, RR, FST, and MWM search strategy data. All other MWM data was analyzed via two-way repeated measure analysis of variance (2W RM ANOVA) for two independent variables (injury status and start position) followed by Holm-Sidak’s *post hoc* test for any significant differences. Weight gain data were analyzed via one-way repeated measure ANOVA for all data points and Student’s t-test for specific time point comparisons. All statistical analyses were performed with SigmaStat 3.5 software by Systat Software, Inc. (San Jose, CA). Significance was set *a priori* at *p*≤0.05.

## Results

### Elevated plus maze

**Fig 1:**
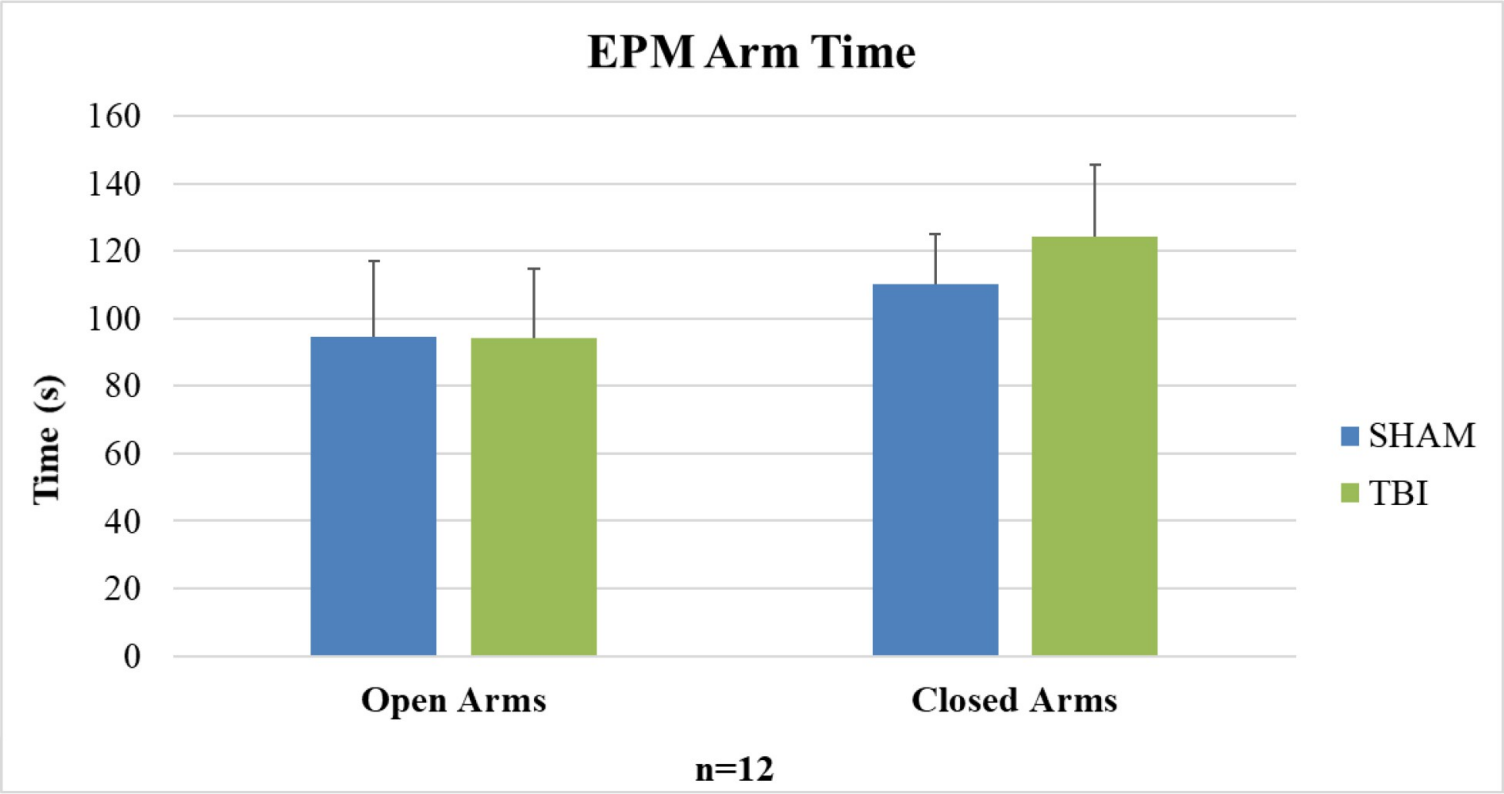
Elevated plus maze arm time. No significant differences in open or closed arm time in the EPM were observed between groups.

Anxiety-like behavior was assessed 14 weeks post-injury. Total time spent in the open and closed arms of the EPM is shown in Fig 1. No significant differences in arm time were observed.

### Rotarod

**Fig 2:**
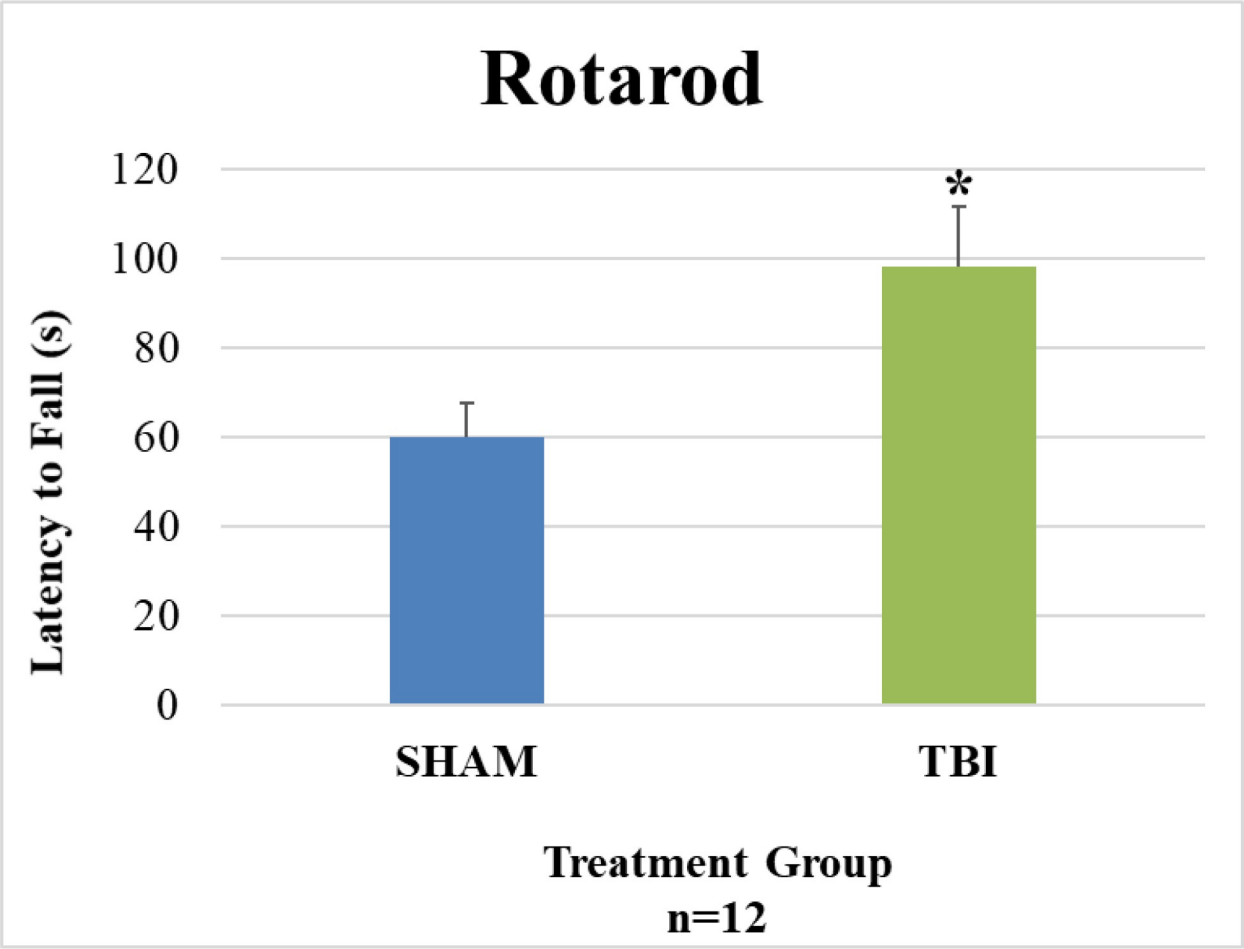
Rotarod latency to fall. TBI animals showed a significantly higher latency to fall than SHAM animals (*p = 0.022).

Motor acuity was evaluated 14 weeks post-injury. Latency to fall in seconds is shown in Fig 2. TBI animals exhibited a significantly higher latency to fall off the rod than SHAM animals (p = 0.022; Student’s t-test).

### Open field test

**Fig 3:**
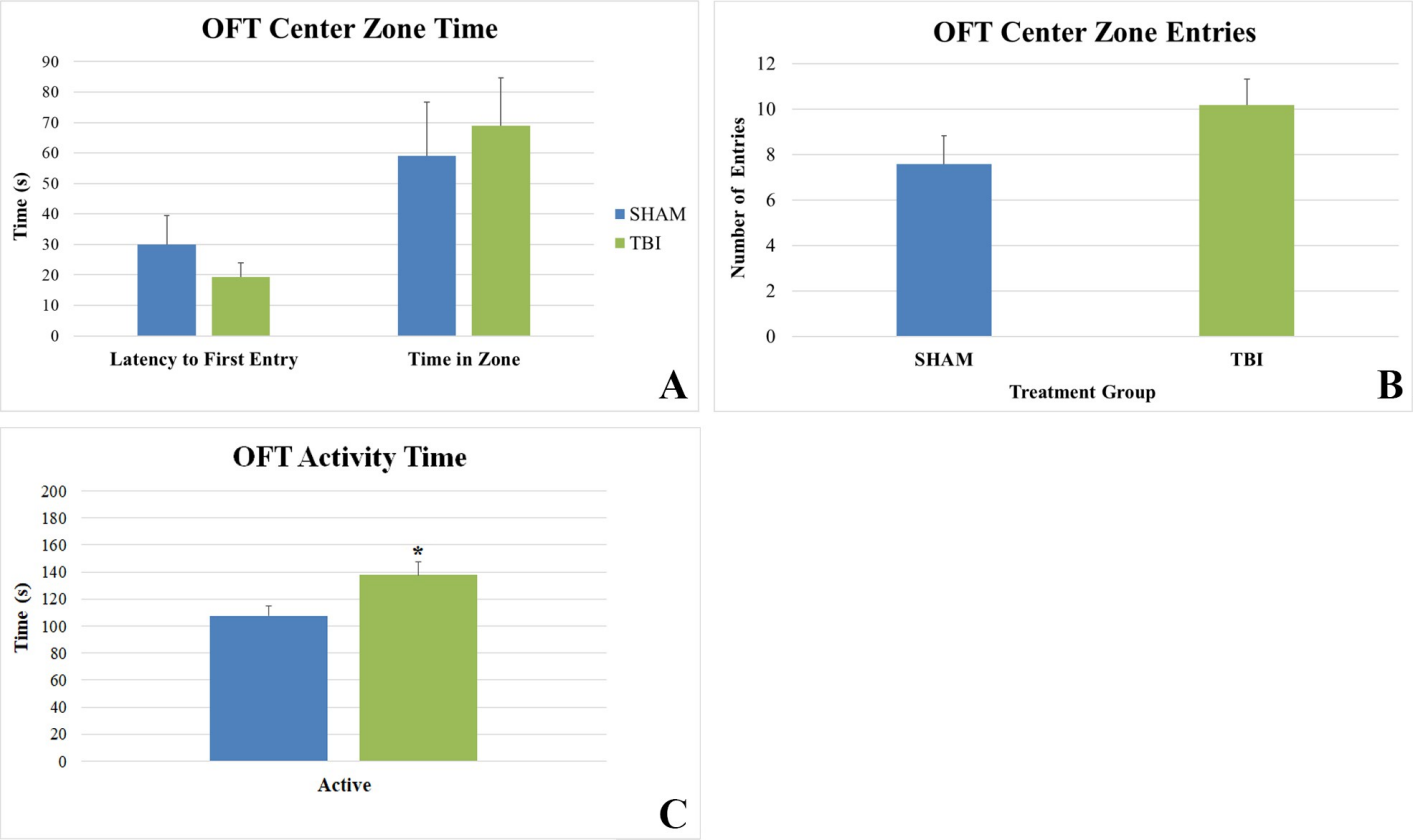
Open field test. A) No differences in latency to first entry or center zone time in the OFT were observed between groups. B) No differences in center zone entries in the OFT were observed between groups. C) TBI animals spent significantly more time active in the OFT (*p = 0.018).

Anxiety-like behavior was also assessed with the OFT 14 weeks post-injury. No differences were observed in latency to first entry, center zone time, or center zone entries (Fig 3A and 3B). However, Fig 3C shows that SHAM animals were significantly less active than TBI animals (p = 0.018; Student’s t-test). The OFT was also used to test general locomotor activity by looking at the distance traveled. No significant difference between groups was observed as the TBI animals traveled 4.48±0.42m and the SHAM animals traveled 4.14±0.47m (mean±SEM).

### Morris water maze

**Fig 4:**
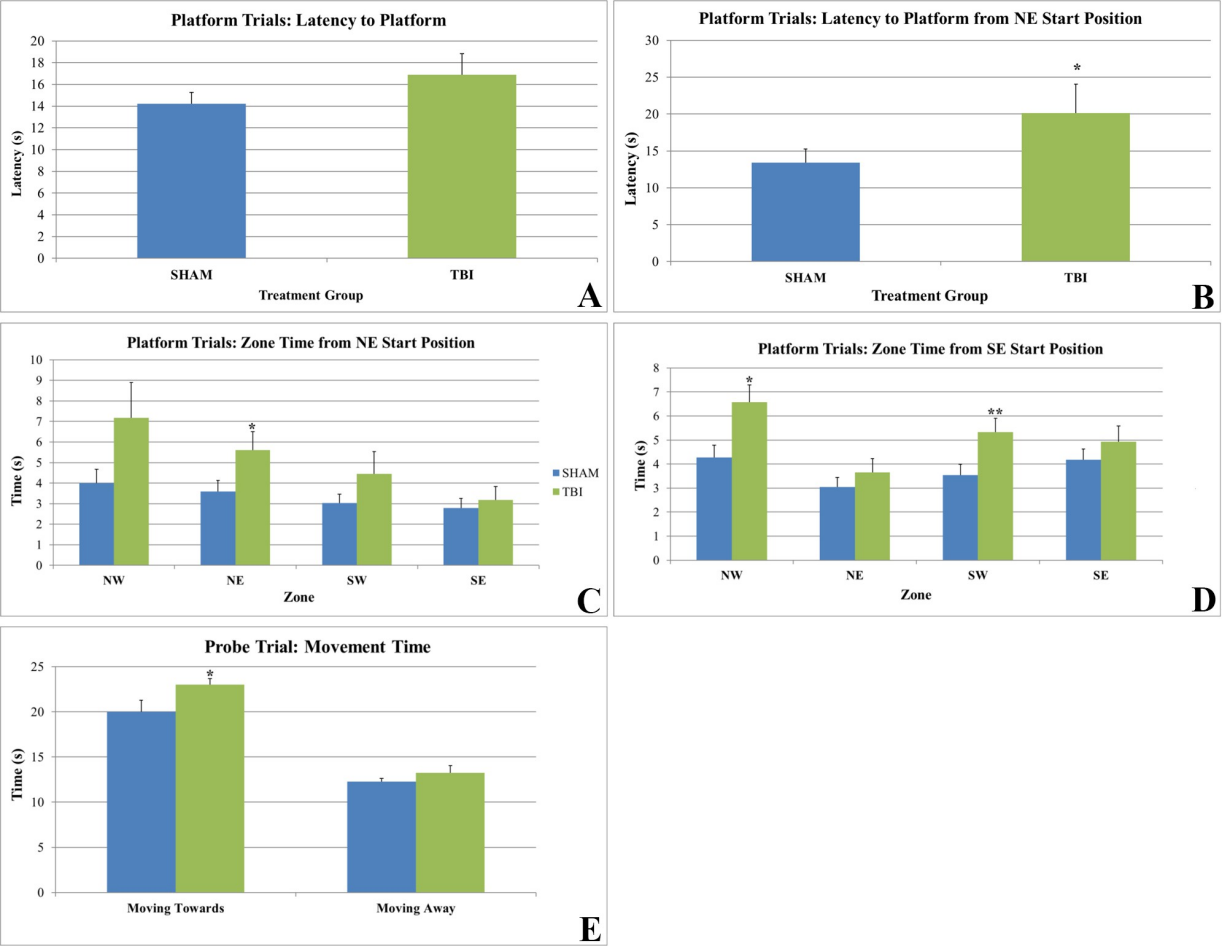
Morris water maze. A) No differences in mean latency to the platform in the MWM were observed between groups. B) TBI animals displayed a significantly higher latency to the platform in the MWM than SHAM animals when beginning the test from the NE start position (*p = 0.041). C) TBI animals spent significantly more time in the NE zone in the MWM than SHAM animals when beginning the test from the NE start position (*p = 0.025). D) TBI animals spent significantly more time in the NW and SW zones in the MWM than SHAM animals when beginning the test from the SE start position (*p = 0.015; **p = 0.024). E) TBI animals spent significantly more time moving towards the platform zone during the probe trial than SHAM animals (*p = 0.050).

Spatial learning and memory were evaluated 15 weeks post-injury. No overall differences between groups were observed in latency to platform, distance traveled, speed, or zone time (Fig 4A). Differences were observed when analyzing each parameter by start position. When starting in the NE zone, TBI animals exhibited a significantly higher latency to the platform (p = 0.041; two-way repeated measure ANOVA; Fig 4B) and spent significantly more time in the NE zone (p = 0.025; two-way repeated measure ANOVA; Fig 4C) than SHAM animals. Additionally, TBI animals spent significantly more time in the NW (p = 0.015; Student’s t test) and SW (p = 0.024, Student’s t-test; Fig 4D) zones when starting in the SE zone. TBI animals also spent significantly more time moving towards the platform zone than SHAM animals during the probe trial (p = 0.050; Student’s t-test; Fig 4E).

### Weight gain

#### Intra-op period

**Fig 5:**
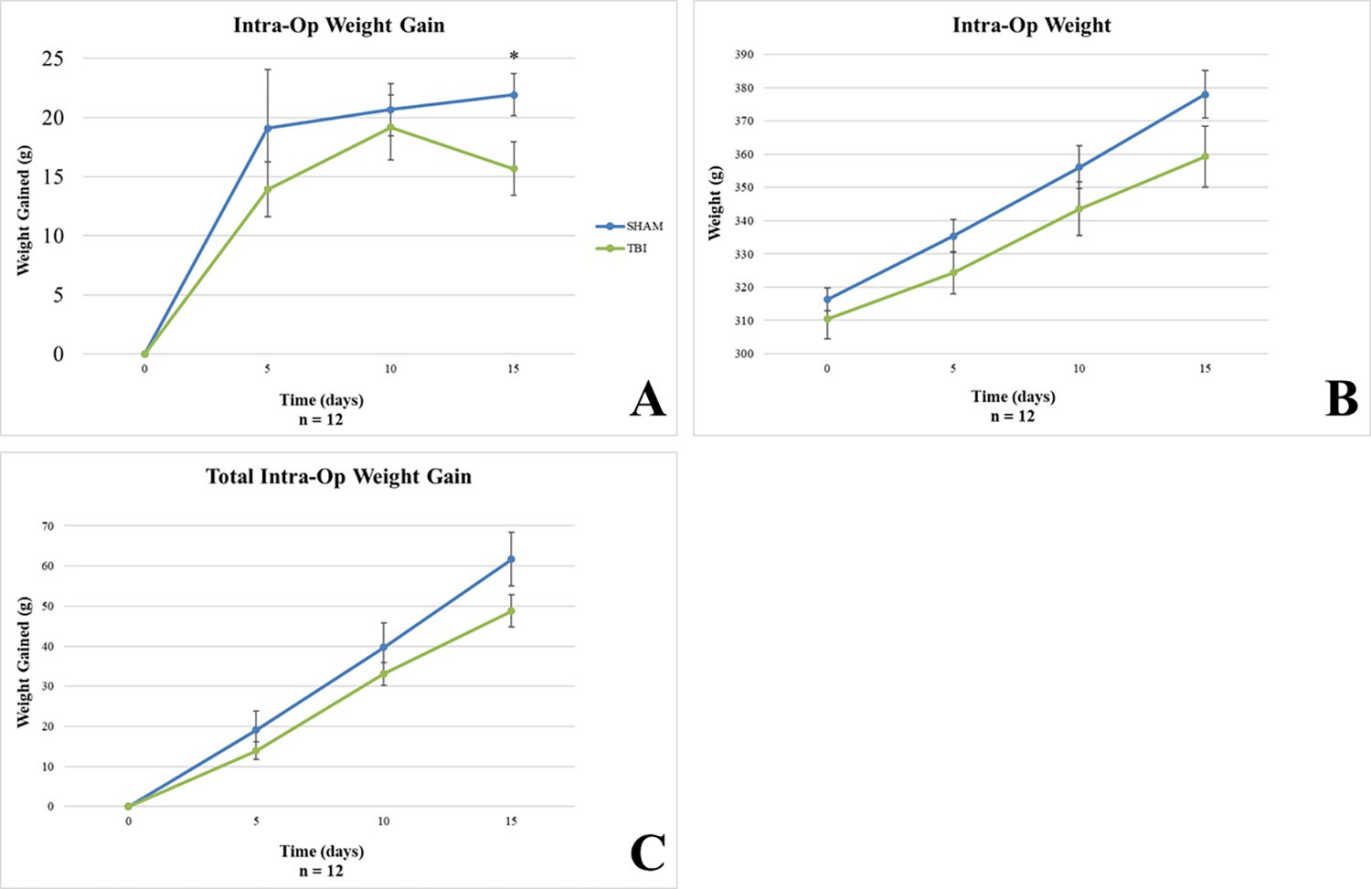
Intra-op weight gain. A) TBI animals gained significantly less weight after the third injury than SHAM animals (*p = 0.041). No differences were observed between groups for B) overall intra-op weight and C) total weight gain.

TBI animals gained significantly less weight than SHAM animals between Intra-Op day 10 and 15 (p = 0.041; Student’s t-test; Fig 5A). No other significant differences between groups were observed in overall weight (Fig 5B), or total weight gained (Fig 5C) during the Intra-Op period.

#### Post-op period

**Fig 6:**
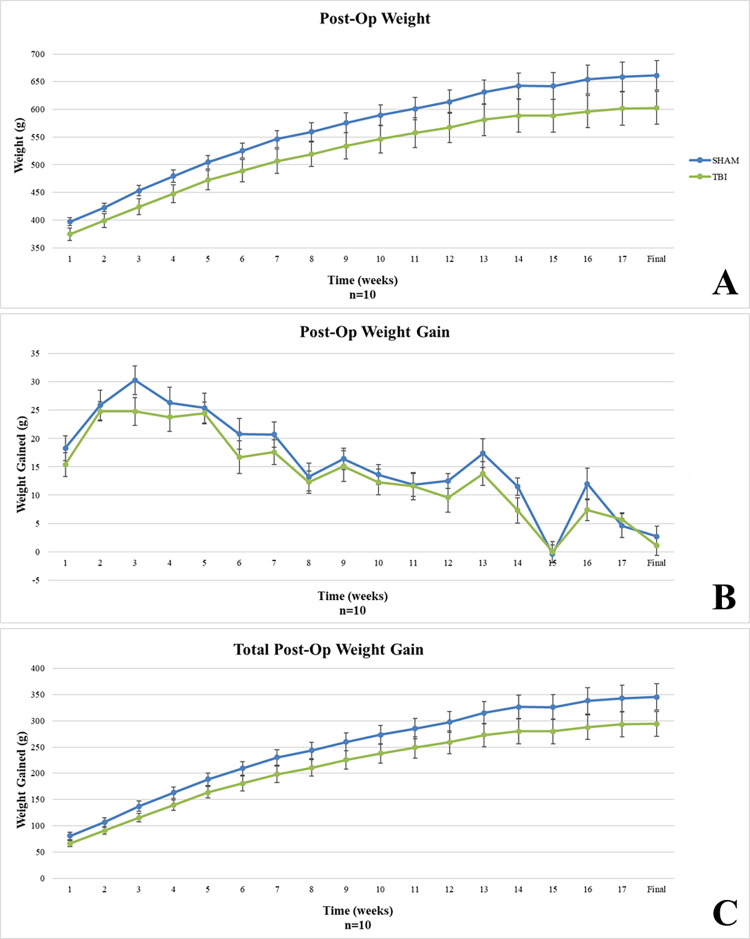
Post-op weight gain. No differences were observed between groups for A) overall post-op weight, B) post-op weight gain per time point, or C) total post-op weight gain.

No significant differences in overall weight (Fig 6A), weight gained per time point (Fig 6B), or total weight gained (Fig 6C) were observed between groups during the Post-Op period.

### RNA-seq analysis

**Fig 7:**
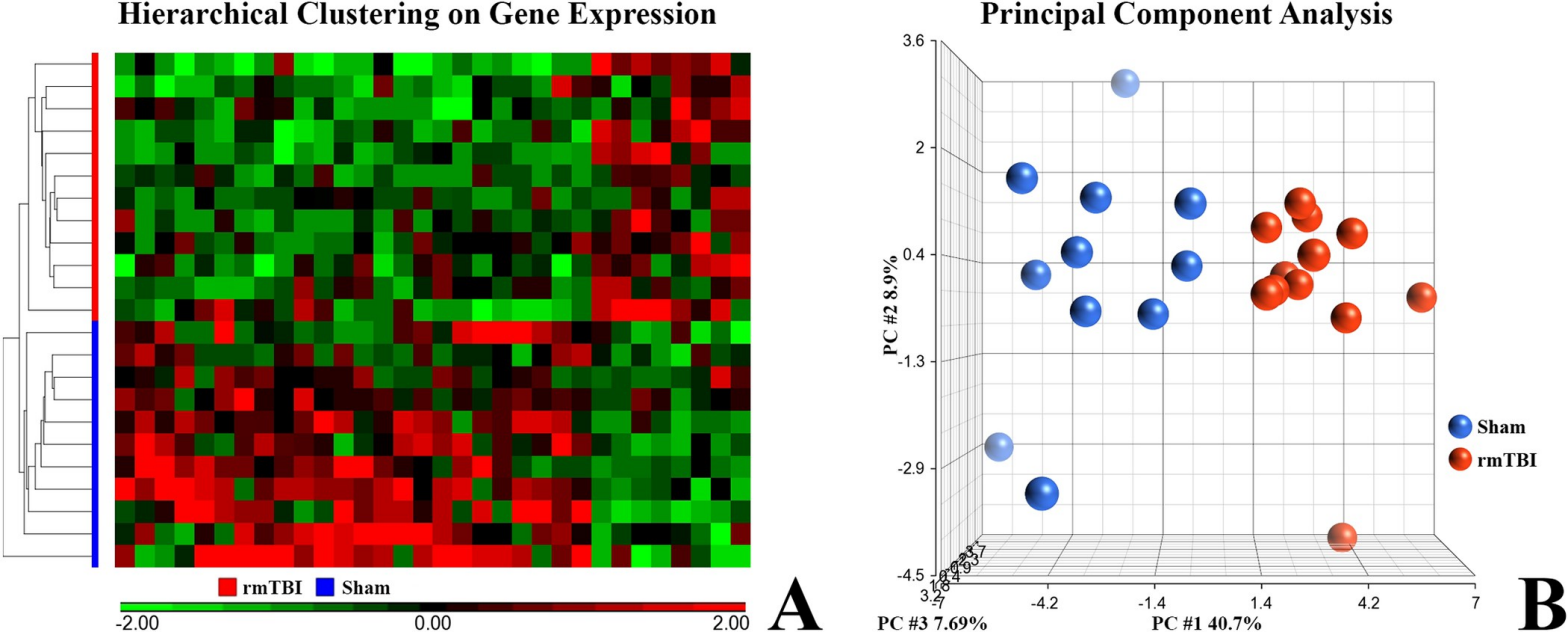
RNA-seq analysis. A) Hierarchical clustering using the resultant panel of genes (each column is a gene) shows distinct clustering by experimental group. B) Principal component analysis also shows a separation of the experimental groups. The first 3 principal components explain 57.3% of the total variance (rmTBI: n = 12; Sham: n = 11).

RNA-seq analysis revealed a genetic fingerprint of the injury that was detected at the chronic endpoint after behavioral testing, demonstrating that rmTBI results in long-lasting alterations in gene expression (Fig 7).

## Discussion

### rmTBI model

We based our rmTBI model on similar models found in the literature [6, 7, 9]. Shultz et al. 2012 and Webster et al. 2015 utilized male Long-Evans rats in repetitive mild lateral fluid percussion (rmLFP) models. While both teams of investigators employed rmLFP, Shultz et al., 2012 animals received one, three, or five injuries, and Webster et al., 2015 animals received three injuries. Conversely, Mouzon et al., 2014 used C57BL/6J mice in a closed head CCI rmTBI model. All three teams of investigators employed behavioral testing paradigms to assess cognitive function and psychological measures (anxiety and depression). Shultz et al., 2012 and Webster et al., 2015 also assessed sensorimotor ability. Behavioral testing schedules varied from eight weeks [6], 12 weeks [9], and 18 months post-injury [7]. Long-term cognitive impairments and psychological deficits were observed in injured animals in all three studies. However, these deficits were most often observed with overt brain damage.

We chose modified injury parameters that we believed would provide a balance between chronic behavioral deficits while minimizing overt damage to the brain. Issues with tissue fixation prevented histological data from being collected for this study. However, gross examination of TBI brains excised after sacrifice showed that no obvious external damage was caused by our model of rmTBI. The limited behavioral deficits observed 14 weeks post-injury could be attributed to the method and parameters our rmTBI model employed. Webster et al., 2015 and Shultz et al., 2012 utilized lateral fluid percussion (LFP) injuries for their respective rat models of rmTBI. It is possible that some results observed from LFP models of rmTBI may not directly translate to CCI models. There could also be a difference between injuries in different rat strains as we used Sprague-Dawley not Long-Evans rats. The CCI model used by Mouzon et al., 2014 was in mice which also may not translate directly to our rat CCI model. Furthermore, our specific rmTBI model may elicit detectable behavioral deficits at earlier post-injury time points that do not persist to the observed chronic behavioral phenotype. Because we have found that performance declines on certain behavioral tasks when repeated, we chose not to do repetitive behavioral testing in this experiment to avoid pre-exposure bias or habituation [12] prior to chronic behavioral testing. Future studies will include earlier behavioral timepoints using tests that can be repeated without bias.

### Behavioral testing

The behavioral assays we selected for this study are standards for measuring depressive-like behavior, anxiety-like behavior, locomotor activity, motor acuity, and spatial learning and memory in rodents. Additionally, these assays are well-documented for having been employed after exposure to models of TBI. While significant differences were not observed in all paradigms, our results indicate the presence of a mild behavioral phenotype.

EPM and OFT data did not indicate any significant change in anxiety-like behavior in our model. These findings were in line with studies using unilateral repetitive mild fluid percussion injury (FPI) models [6, 9]. Only when severe anatomical damage was observed did anxiety like behavior increase [6]. Krukowski et al. and Mouzon et al. reported exposure to rmTBI significantly increased EPM open arm time in male mice [13, 14] but these studies used closed-head injury models instead of open-head injuries like CCI and FPI. The lack of anxiety-like behavioral changes in our model may be attributable to our model being less severe or that a more diffuse injury, like closed-head injury, may be necessary to see an effect.

TBI animals having a significantly higher latency to fall from the rod than SHAM in the RR which was an unexpected result. We also found that TBI animals were more active than SHAM in the OFT and there was no difference in distance traveled. Both results indicate that the TBI animals’ gross locomotor performance is similar or better when compared to SHAM. In a mouse closed-head model of TBI, Homsi et al. 2009 demonstrated locomotor hyperactivity, locomotion and rearing, up to 12 weeks post-injury [15]. The mechanism underlying this hyperactivity is unclear and may not be generalizable to our CCI model. It may be that the five-day time interval between injuries is activating a potential neuroprotective mechanism, preconditioning, that is revealed by this motor task. Such a mechanism was demonstrated by Allen et al. 2000 where successive mild injuries did not affect motor performance and even prevented a subsequent severe injury from having a behavioral effect [16]. Determining whether this mechanism is at play in our model would require further study. Another possibility is that we are observing complete recovery of function after rmTBI. Shultz et al. 2012 did not see any differences on motor tasks 8 weeks post-injury even with a more severe injury paradigm [6]. However, Webster et al. did report motor deficits 12 weeks after injury [9]. Tucker et al. suggests that 1-month post-injury may be the upper limit for studying behavioral deficits after mild TBI [12]. There may also be a lack of motivation for SHAM animals to continue the task. The main motivating factor for staying on the rod is fear of falling. Our RR apparatus had a fall height of approximately 18 inches which is typical for mice but may not be a significant deterrent for our large rats. Other rotarod apparatuses have fall heights up to 48 inches for rats. Whether a higher fall height would alter the observed results is unknown.

TBI animals did not show an overall higher latency to the platform than SHAM animals in the MWM but spent more time moving towards the platform zone during the probe trial. The unilateral injury employed in the rmTBI model used in this study could have potentially caused an imbalance in visual acuity. Morriss et al. 2021 demonstrated that rmTBI, albeit closed head injury in mice, resulted in impaired visual acuity and reduced optic nerve diameter and concluded that visual dysfunction was the main determinant of poor MWM performance [17]. In another mouse model of closed head rmTBI, Pinkowski et al. 2020 reported impairment of visual discrimination learning [18]. Therefore, the significantly higher latency to the platform observed specifically when starting the test from the NE zone could be vision-related, in addition to an impairment in spatial memory and learning. Future studies could utilize strains of animals known to possess functional eyesight (i.e., Long-Evans rats) and assays for evaluating visual competency to determine if this is a factor in this injury model.

### Weight gain

In our model, rmTBI seems to have an increasing negative effect on weight gain during the intra-op period. This effect may be attributed to the stress and pain of the surgical procedures and repeated injuries. The lack of differences observed during the post-op period likely resulted from the high level of variability within each group, which may have been caused by differing injury recovery patterns between groups and stress from behavioral testing after 14 weeks post-injury.

Anthropometric measures such as weight gain/loss are key standards of health and wellness assessment and can be used to indicate recovery milestones following injury in humans. While this data is routinely collected, there is a remarkable lack in the evaluation of the implications of these measures in TBI research, particularly in preclinical models. Other investigators have reported weight loss with similar weight drop models. Interestingly, this weight loss observation has been shown to be consistent over several models of experimental TBI including controlled cortical impact (CCI) [19], lateral and midline fluid percussion injury (FPI) [20–23], weight drop [24–26], and blast injury [27, 28]. Most of the observations have been reported within the first 3 days post-injury but a couple of studies have shown longer lasting weight deficits (Lapinlampi et al., 2020; Moinard et al., 2005).

Some of the TBI studies that report weight data also suggest mechanisms for the observed weight deficits including poor appetite, changes in leptin levels, acute stress, and nonconvulsive status epilepticus. Poor appetite resulting in less food intake in the days following injury could explain the weight loss [25, 29]. But one study demonstrated that the nutritional state of the animal is actually altered by TBI including distal intestinal atrophy and other metabolic alterations [22]. This indicates that poor appetite alone does not explain the data. Leptin, when present, can decrease appetite and increase energy consumption [20] and leptin levels increase in the early post-injury period after moderate TBI [30]. However, Leptin levels decrease after severe TBI [30], which does not explain increased weight loss with increasing injury severity. The weight data could be linked to acute stress caused by TBI [26, 28]. We have seen weight loss associated with some of our more stressful behavioral tests and objective indicators of stress (e.g., corticosterone) seem to rise at the time of post-TBI weight loss [28]. But corticosterone levels can remain elevated for extended periods of time—well beyond the period of acute stress and weight loss [23]. Lapinlampi et al. suggest that nonconvulsive status epilepticus lasting 3–4 days post-injury may be a contributing factor to the observed weight loss [21] but this seems to occur only with severe injuries [31]. Ultimately, at this point, there does not seem to be a clear-cut mechanism to explain the weight data observed post-TBI. The results of this study demonstrate the need to fill this knowledge gap by analyzing trends in weight gain/loss during and after the injury period.

### RNA-seq analysis

The finding that gene expression changes persist into chronic survival time points despite the mild behavioral phenotype observed demonstrates the potential for using sequencing to improve diagnosis of chronic neurological deficits. One could envision using the panel of genes, or molecular signature, identified in this study to “diagnose” unknown rat injuries bearing the same molecular signature. Comprehensive diagnostic potential would require the building of a library of diagnostic gene panels for which the injury parameters are known.

### Translational perspectives

The mild memory deficits we observed with our chronic behavioral tests have correlates in studies of human concussion and mTBI. When compared to controls with mild non-head injuries, mTBI patients/participants had memory deficits that persisted 3 [32–34], 6 [35], and 12 [34] months post-injury. Further, the Immediate Post-Concussion Assessment and Cognitive Testing (ImPACT) Post-Concussion Symptom Scale (PCSS) has a visual memory composite score [36] and this is the specific memory measure that persists the longest post-injury [32, 33]. This correlates well with the potential visual acuity imbalance affecting our memory task which has a visual component.

The potential of RNA-seq as an effective diagnostic tool for brain injury has also been demonstrated in human studies. Meller and colleagues showed that RNA-seq can identify characteristic expression patterns of exons, or a molecular signature, in blood samples drawn from stroke and TBI patients that can distinguish the injured patients from controls [37, 38]. A strength of this technique is to also detect other information coded into the gene expression patterns including age range, gender, and the number of mTBIs. Translation of this technology would require the involvement of populations with a high prevalence of TBI like Veterans or TBI clinics and centers to build a significant library of validated molecular signatures that could be used diagnostically. This would be a significant undertaking which is why single or predefined panels of blood biomarkers are currently preferred over this unbiased analysis of gene expression.

## Study limitations

This study had a few issues that resulted in reduced impact of the stated findings. 1) Issues with tissue fixation prevented histological data from being collected. Anatomical confirmation of the injury severity would have supported the behavioral and RNA-seq data presented. 2) FST data would have provided additional information about the psychological state of the animals, but the cylinders were not large enough for the rats in this study. 3) Collection of behavioral data at earlier timepoints on tasks that are repeatable without bias would have provided insight into whether behavioral deficits were present early and recovered over time. This will be important to include in future studies.

## Conclusions

Overall, the parameters chosen for our rmTBI model have shown to not elicit the robust chronic behavioral deficits that we hypothesized would be observed 14 weeks post-injury. However, we did observe a mild behavioral phenotype caused by this injury that significantly affected performance on a spatial learning and memory task (MWM). Some increased locomotor activity was also observed in TBI animals. Chronic anxiety-like behavior was not changed by our rmTBI model. Adjustments to and optimization of this injury model need to be implemented in future studies to achieve long-term chronic behavioral deficits and comprehensive significant differences between groups. The results of this study also suggest that anthropometric measures, including weight gain, should be monitored closely immediately following TBI to indicate recovery from injury. The potential diagnostic power of RNA-seq analysis was demonstrated with gene expression at the chronic endpoint distinguishing between rmTBI and Sham despite the mild behavioral phenotype.
